# Diagnostic Accuracy of C-reactive Protein, Procalcitonin, White Blood Cell Count, and Neutrophil-Lymphocyte Ratio in the Early Detection of Post-surgical Infections: A Systematic Review

**DOI:** 10.7759/cureus.81853

**Published:** 2025-04-07

**Authors:** Mawada Taha, Usama Shafique, Wardah Rashid, Hussein Taha, Manahil Awan, Aisha Ayyub, Shahzad Ahmad, Lara Alsadoun

**Affiliations:** 1 General Surgery, National Ribat University, Khartoum, SDN; 2 General Surgery, Azra Naheed Medical College, Lahore, PAK; 3 Internal Medicine, Khawaja Muhammad Safdar Medical College, Lahore, PAK; 4 Surgery, Nahda College, Khartoum, SDN; 5 General Practice, Liaquat National Hospital, Karachi, PAK; 6 Pathology, Sir Syed College of Medical Sciences for Girls (SSCMS), Karachi, PAK; 7 Cardiac Surgery, Liaquat National Hospital, Karachi, PAK; 8 Trauma and Orthopaedics, Chelsea and Westminster Hospital, London, GBR

**Keywords:** biomarker accuracy, c-reactive protein, neutrophil-lymphocyte ratio, post-surgical infection, procalcitonin, white blood cell count

## Abstract

Early detection of post-surgical infections is crucial for improving patient outcomes and reducing healthcare burdens. This systematic review evaluates the diagnostic accuracy of C-reactive protein (CRP), procalcitonin (PCT), white blood cell count (WBC), and neutrophil-lymphocyte ratio (NLR) in identifying early post-surgical infections across various surgical specialties. A comprehensive search was conducted in PubMed, MEDLINE, Embase, and Cochrane Library following Preferred Reporting Items for Systematic Reviews and Meta-Analyses (PRISMA) guidelines, yielding eight high-quality studies, including meta-analyses, randomized controlled trials, and cohort studies. The findings indicate that CRP is the most extensively studied biomarker, with postoperative day (POD) 3-7 levels demonstrating moderate to high predictive value in abdominal, colorectal, spinal, and pancreatic surgeries. PCT was effective in guiding the management of adhesion-related small bowel obstruction, while NLR showed moderate diagnostic performance in orthopedic infections. Sensitivity and specificity varied across biomarkers and surgical types, with CRP showing the highest accuracy in spinal surgery (100% sensitivity and 96.8% specificity). Quality assessment using AMSTAR 2, ROB 2, QUADAS-2, and NOS tools revealed a moderate risk of bias in most studies due to heterogeneity in methodologies and biomarker cutoffs. The results support the integration of biomarker-based infection monitoring into perioperative protocols to optimize patient management, facilitate early discharge, and reduce unnecessary antibiotic use. Future research should focus on large-scale multicenter trials to establish standardized biomarker thresholds and explore the potential of combining multiple biomarkers with artificial intelligence-driven predictive models.

## Introduction and background

Post-surgical infections remain a significant concern in clinical practice, contributing to increased morbidity, prolonged hospital stays, and higher healthcare costs [1]. These infections, often categorized under surgical site infections (SSIs) and postoperative systemic infections, can arise due to various factors, including preexisting patient conditions, intraoperative contamination, and delayed postoperative monitoring [2]. Early and accurate detection of infections is crucial to ensuring timely intervention, reducing complications, and improving patient outcomes [3]. Traditionally, clinical assessments, imaging modalities, and laboratory markers have been utilized to identify early signs of infections. Among the laboratory-based methods, white blood cell (WBC) count, C-reactive protein (CRP), and procalcitonin (PCT) have emerged as essential biomarkers in assessing postoperative inflammatory responses and distinguishing between normal post-surgical inflammation and infectious complications [4].

CRP, an acute-phase protein produced by the liver in response to inflammation, has been widely studied as a diagnostic tool for detecting post-surgical infections [5]. Its levels tend to rise within six to 12 hours postoperatively, peaking within 48 hours, and typically decline thereafter unless an infectious process is present. However, its specificity in differentiating infectious versus non-infectious inflammatory responses remains a challenge, necessitating the integration of additional biomarkers [6]. Procalcitonin, a precursor of calcitonin, has gained prominence as a more specific marker for bacterial infections, as its levels tend to rise rapidly in response to systemic infections but remain relatively stable in cases of non-infectious inflammatory responses [7]. While WBC count has long been a routine indicator of infection, it often lacks specificity due to its tendency to rise postoperatively in response to surgical trauma rather than infection alone.

To systematically evaluate the diagnostic accuracy of CRP, PCT, and WBC in detecting early post-surgical infections, we structured this systematic review based on the PICO (Population, Intervention, Comparison, and Outcome) framework [8]. Our Population (P) includes post-surgical patients who are at risk of developing infections following major surgical procedures. The Intervention (I) consists of laboratory-based biomarker assessments (CRP, PCT, and WBC) for early detection of infection. The Comparison (C) involves alternative diagnostic methods, including physical examination findings such as pallor, jaundice, cyanosis, or edema. Along with this, signs and symptoms include fever, dyspnea, chest pain, altered mental status, discharge, fever, or neurological deficits.

Other laboratory markers are routine blood tests (e.g., CBC, CRP, electrolytes), imaging studies, and other biochemical parameters. Finally, the Outcome (O) of interest is the diagnostic accuracy of these biomarkers in differentiating infectious versus non-infectious post-surgical inflammatory responses, particularly in terms of sensitivity, specificity, and predictive value. Through this systematic review, we aim to synthesize the current evidence regarding the effectiveness of these biomarkers in improving early detection, guiding treatment decisions, and ultimately enhancing post-surgical patient care.

## Review

Materials and methods

Search Strategy

The search strategy for this systematic review followed the Preferred Reporting Items for Systematic Reviews and Meta-Analyses (PRISMA) guidelines [9], ensuring a comprehensive and transparent selection process. A structured search was conducted across PubMed, MEDLINE, Embase, and Cochrane Library, using a combination of MeSH terms and keywords related to CRP, PCT, WBC, NLR, SSIs, and postoperative infections. Boolean operators (AND/OR) were applied to refine search queries and retrieve relevant studies from the last two decades. Studies were screened based on predefined eligibility criteria, including RCTs, cohort studies, systematic reviews, and meta-analyses evaluating biomarker accuracy in detecting early post-surgical infections. Titles and abstracts were independently reviewed by two authors, followed by a full-text assessment of selected articles. Data extraction and quality assessment were performed using validated tools such as AMSTAR 2, ROB 2, QUADAS-2, and the Newcastle-Ottawa Scale (NOS) to minimize bias and enhance reliability. The PRISMA flow diagram was used to illustrate the study selection process, ensuring clarity in identifying included and excluded studies, thus reinforcing the robustness and reproducibility of this review.

Eligibility Criteria

The eligibility criteria for this systematic review were defined to ensure the inclusion of high-quality, clinically relevant studies assessing the diagnostic accuracy of CRP, PCT, WBC, and NLR in detecting early post-surgical infections. Studies were included if they were randomized controlled trials (RCTs), cohort studies, systematic reviews, or meta-analyses that investigated the role of these biomarkers in surgical patients. Only studies that provided quantitative measures of sensitivity, specificity, or predictive values were considered, ensuring a robust analysis of biomarker performance. The review focused on adult surgical populations undergoing major procedures, including abdominal, orthopedic, colorectal, spinal, and hepatopancreatobiliary surgeries, where infection risk is a significant postoperative concern. Exclusion criteria included case reports, conference abstracts, editorials, non-English publications, studies lacking full-text availability, and those focusing on pediatric or non-surgical populations. Studies with insufficient data, unclear diagnostic criteria, or lacking biomarker-specific outcomes were also excluded to maintain methodological rigor. The selection process prioritized studies with clearly defined infection criteria, standardized biomarker cut-offs, and appropriate follow-up durations to ensure the findings were clinically applicable and generalizable to real-world surgical settings.

Data Extraction

Data extraction was conducted systematically to ensure consistency, accuracy, and reproducibility in analyzing the included studies. A standardized data extraction form was developed to collect key study characteristics, including author names, publication year, study design, sample size, surgical type, biomarkers assessed (CRP, PCT, WBC, and NLR), main findings, sensitivity, specificity, and overall conclusions. Two independent reviewers extracted data, and discrepancies were resolved through discussion or consultation with a third reviewer to minimize bias. Specific attention was given to the cut-off values used for biomarkers, the timing of post-surgical measurements, and the reported diagnostic accuracy metrics to facilitate direct comparisons across studies. Where applicable, additional statistical details such as area under the curve (AUC), likelihood ratios, and predictive values were documented to enhance the interpretation of biomarker efficacy. The extracted data were then organized into structured tables to provide a clear synthesis of findings, ensuring that comparisons between different surgical types and biomarkers were feasible. In cases where relevant information was missing or unclear, the corresponding authors of the original studies were contacted for clarification. This structured approach ensured that the review maintained a high level of methodological rigor, allowing for reliable conclusions regarding the diagnostic accuracy of biomarkers in early post-surgical infection detection.

Data Analysis and Synthesis

Data analysis and synthesis were conducted using a structured approach to evaluate the diagnostic accuracy of CRP, PCT, WBC, and NLR in detecting early post-surgical infections across various surgical specialties. Extracted data were systematically reviewed, focusing on biomarker sensitivity, specificity, predictive values, and AUC from receiver operating characteristic (ROC) analyses. Studies were grouped based on the surgical type and biomarker assessed to identify trends and patterns in diagnostic performance. A qualitative synthesis was performed to compare findings across studies, highlighting consistent biomarker cut-off thresholds, trends in postoperative biomarker kinetics, and differences in diagnostic performance based on surgical procedure and patient population. Where possible, variations in results due to heterogeneity in study designs, biomarker measurement timing, and infection definitions were analyzed to assess potential sources of bias. In addition, risk-of-bias assessment tools such as AMSTAR 2 [10], ROB 2 [11], QUADAS-2 [12], and the Newcastle-Ottawa Scale [13] were applied to assess study quality and reliability. The synthesized findings were then contextualized within existing clinical guidelines and prior literature, ensuring a comprehensive evaluation of how biomarker-based infection monitoring can optimize surgical infection management and patient outcomes.

Results

Study Selection Process

**Figure 1:**
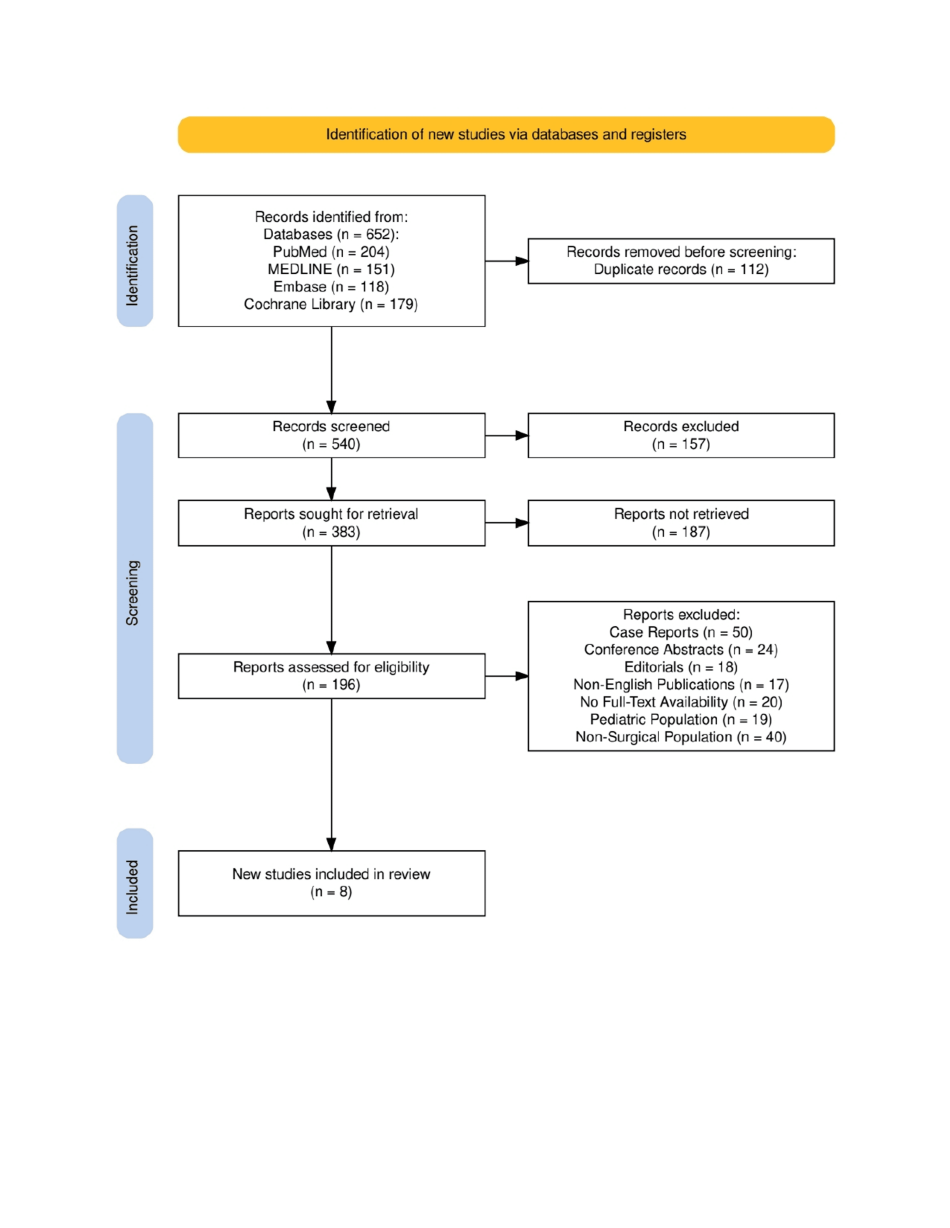
The Preferred Reporting Items for Systematic Reviews and Meta-Analyses (PRISMA) flowchart represents the study selection process.

The study selection process followed the PRISMA guidelines, as illustrated in Figure 1. A total of 652 records were identified across four major databases (PubMed, MEDLINE, Embase, and Cochrane Library). After the removal of 112 duplicate records, 540 studies were screened based on titles and abstracts. Following the initial screening, 157 studies were excluded, and 383 full-text reports were sought for retrieval, of which 187 could not be retrieved due to various reasons, such as lack of full-text availability or restricted access. The remaining 196 studies were assessed for eligibility, with 188 reports excluded for not meeting the predefined inclusion criteria. Excluded studies consisted of 50 case reports, 24 conference abstracts, 18 editorials, 17 non-English publications, 20 studies without full-text availability, 19 pediatric-focused studies, and 40 non-surgical studies. Ultimately, eight studies were included in the final systematic review, ensuring a rigorous and methodologically sound selection process that enhances the reliability and clinical relevance of the findings.

Characteristics of the Selected Studies

The characteristics of the selected studies, as summarized in Table 1, highlight the diverse range of surgical specialties, study designs, and biomarker evaluations included in this systematic review. The studies encompassed meta-analyses, clinical trials, cohort studies, and RCTs, ensuring a comprehensive assessment of biomarker utility across different surgical procedures. Sample sizes varied widely, ranging from 180 to 3,737 patients, with a focus on abdominal, colorectal, spinal, pancreatic, orthopedic, and adhesion-related small bowel obstruction (ASBO) surgeries. Among the biomarkers analyzed, CRP was the most extensively studied, demonstrating high diagnostic accuracy on postoperative days (POD) 3-7 across multiple studies, particularly in abdominal, spinal, and pancreatic surgeries. PCT was assessed in ASBO management, where its implementation significantly improved clinical decision-making by reducing hospital stay and time to surgery. NLR was evaluated in orthopedic procedures, showing moderate diagnostic value with an AUC of 0.80, although its predictive performance varied depending on surgical type and infection classification. While several studies established high sensitivity and specificity for CRP, particularly in spinal surgery, other findings suggested that elevated CRP alone may not always lead to earlier infection detection, as seen in colorectal procedures. Preoperative biomarker trends were also explored, with some studies identifying CRP, albumin, and eGFR as potential predictors of surgical site infection risk in elective procedures. Despite some heterogeneity in biomarker thresholds and study methodologies, the findings collectively support the use of CRP, PCT, and NLR as valuable tools for early post-surgical infection monitoring, with implications for tailored postoperative management strategies based on specific surgical contexts. 

**Table 1:** Characteristics of the selected studies AUC: area under the curve, ASBO: adhesion-related small bowel obstruction, CRP: C-reactive protein, CT: computed tomography, DOR: diagnostic odds ratio, eGFR: estimated glomerular filtration rate, NPV: negative predictive value, NLR: neutrophil-lymphocyte ratio, PCT: procalcitonin, POD: postoperative day, ROC: receiver operating characteristic, RCT: randomized controlled trial, SSI: surgical site infection, WBC: white blood cell count

Study	Study type	Sample size	Surgical type	Biomarkers sssessed (CRP/PCT/WBC)	Main findings	Sensitivity (%)	Specificity (%)	Conclusion
Adamina et al., 2015 [14]	Meta-analysis	1986	Abdominal surgery (gastric bypass, colectomy, esophageal, pancreatic, gastric, colorectal resections)	CRP	CRP levels on post-op day 4 had the highest diagnostic accuracy (AUC = 0.76). Elevated CRP correlated with post-surgical infections.	68.5%	71.6%	CRP on day 4 is a reliable tool for ruling out infections due to its high negative predictive value (84.3%).
Plat et al., 2021 [15]	Clinical trial	350	Major abdominal surgery	CRP	CRP levels on post-op days 3, 4, and 5 correlated with major infectious complications. Cut-off values: 175 mg/L (day 3), 130 mg/L (day 4), 144 mg/L (day 5). Negative predictive value (NPV) for discharge was 97%.	80%	65%	CRP cut-offs can guide safe discharge decisions and identify high-risk patients needing intervention.
Peng et al., 2024 [16]	Systematic review and meta-analysis	3,737	Orthopedic surgery	Neutrophil-Lymphocyte Ratio (NLR), CRP, WBC	NLR showed moderate accuracy in detecting post-op infections. AUC = 0.80, DOR = 7.76. Variability in results due to surgical type and infection classification.	77%	69%	NLR is useful for infection prediction but should be combined with other biomarkers for better diagnostic accuracy.
Singh et al., 2024 [17]	Clinical trial	180	Colorectal surgery	CRP	Elevated CRP on POD 3-5 (cut-offs: 170 mg/L on POD3, 125 mg/L on POD4) was not associated with earlier infection detection. Investigating rising CRP led to more CT scans but did not improve outcomes.	N/A	N/A	Routine CRP monitoring helps rule out infections, but investigating elevated CRP alone does not lead to earlier diagnosis.
Mujagic et al., 2018 [18]	Randomized controlled trial (RCT) - observational cohort study	2093	General surgery (elective procedures)	CRP, hemoglobin, albumin, eGFR	Preoperative CRP levels were significantly associated with higher odds of surgical site infection (SSI). Albumin and eGFR levels also influenced infection risk. ROC AUC for albumin = 0.62.	N/A	N/A	Preoperative CRP may help assess SSI risk, but delaying elective surgery based on biomarker levels is not generally recommended.
Cossé et al., 2017 [19]	Retrospective cohort study (before-after study design)	163	Adhesion-related small bowel obstruction (ASBO)	Procalcitonin (PCT)	Implementing a PCT-based algorithm significantly improved appropriate ASBO management (86% vs. 47%, p<0.001), reduced time to surgery (48h vs. 72h, p=0.02), and shortened length of hospital stay (4 vs. 6 days, p=0.02) without increasing morbidity or mortality.	N/A	N/A	PCT-based algorithm is effective in optimizing ASBO management, reducing hospital stay and time to surgery without additional surgical burden.
Kang et al., 2010 [20]	Clinical trial	348	Spinal surgery (decompression, extensive procedures)	CRP	CRP levels followed a characteristic pattern in 95.4% of patients. A continuous or second rise in CRP on POD 5-7 indicated a high risk of infection. Early intervention with antibiotics based on CRP trends prevented severe complications.	100%	96.8%	CRP screening is a simple and reliable method for early detection of post-spinal surgery infections, with high predictive accuracy.
Warschkow et al., 2012 [21]	Meta-analysis and diagnostic study	280 (Meta-analysis included additional studies)	Pancreatic surgery	CRP	CRP levels had the highest diagnostic accuracy on POD 7 (AUC = 0.77). On POD 4, AUC was 0.67. Pooled sensitivity was 63% on POD 4 and 75% on POD 6, while specificity was 79% on POD 4. Considerable heterogeneity was observed.	63% (POD 4), 75% (POD 6)	79% (POD 4)	CRP has moderate diagnostic accuracy for detecting inflammatory complications post-pancreatic surgery, with peak accuracy on POD 6-7. Larger studies are needed for better precision.

Quality Assessment

The quality assessment of the included studies was conducted using validated assessment tools tailored to each study design, ensuring a transparent evaluation of methodological rigor and potential biases (Table 2). The meta-analyses were assessed using AMSTAR 2, where studies demonstrated a moderate risk of bias, primarily due to heterogeneity in methodologies and lack of protocol registration, although systematic study selection and minimal publication bias strengthened their reliability. The clinical trials were evaluated using the ROB 2 tool, with findings indicating a low to moderate risk of bias. While most trials were well-designed and randomized and had appropriate statistical analyses, some had unclear blinding procedures and allocation concealment. The RCT with an observational cohort component was assessed using ROBINS-I, revealing a moderate risk of bias due to potential confounding factors, although statistical adjustments were implemented to mitigate these concerns. The retrospective cohort study was evaluated using the NOS, where limitations in controlling confounding variables were noted, but adequate follow-up and well-structured comparability between groups improved the study’s credibility. The diagnostic accuracy study and meta-analysis were assessed using QUADAS-2, showing a moderate risk of bias, largely due to variations in biomarker measurement methodologies across included studies. Overall, while some studies exhibited limitations in study design, blinding, and heterogeneity, the majority adhered to high methodological standards, allowing for a comprehensive and credible synthesis of biomarker accuracy in post-surgical infection detection.

**Table 2:** Evaluation of methodological rigor and potential biases AMSTAR 2: Assessing the Methodological Quality of Systematic Reviews (second version), NOS: Newcastle-Ottawa Scale, QUADAS-2: Quality Assessment of Diagnostic Accuracy Studies (second version), RCT: randomized controlled trial, ROB 2: Cochrane Risk of Bias tool for Randomized Controlled Trials, ROBINS-I: Risk of Bias in Non-Randomized Studies of Interventions

Study	Study type	Assessment tool used	Risk of bias (low/moderate/high)	Key quality assessment findings
Adamina et al., 2015 [14]	Meta-analysis	AMSTAR 2 (Assessing the Methodological Quality of Systematic Reviews)	Moderate	Lacked a protocol registration, heterogeneity was moderate, and study selection was comprehensive.
Plat et al., 2021 [15]	Clinical trial	ROB 2 (Cochrane Risk of Bias for RCTs)	Low	Well-designed and randomized and included appropriate statistical analysis, but blinding was not explicitly mentioned.
Peng et al., 2024 [16]	Systematic review and meta-analysis	AMSTAR 2	Moderate	Some heterogeneity was observed, but study selection was systematic, and publication bias was minimal.
Singh et al., 2024 [17]	Clinical trial	ROB 2	Moderate	Allocation concealment and blinding were unclear, but outcome measures were robust.
Mujagic et al., 2018 [18]	Randomized controlled trial (RCT) - observational cohort study	ROBINS-I (Risk of Bias in Non-Randomized Studies of Interventions)	Moderate	Observational design introduced some confounding factors; however, statistical adjustments were made to minimize bias.
Cossé et al., 2017 [19]	Retrospective cohort study	Newcastle-Ottawa Scale (NOS)	Moderate	The retrospective nature limited control over confounding, but follow-up and comparability between groups were well-executed.
Kang et al., 2010 [20]	Clinical trial	ROB 2	Low	Well-controlled study with robust statistical analysis, although some aspects of blinding were not reported.
Warschkow et al., 2012 [21]	Meta-analysis and diagnostic study	QUADAS-2 (Quality Assessment of Diagnostic Accuracy Studies)	Moderate	Some studies included had variations in methodologies, leading to moderate risk of bias, but overall well-conducted.

Discussion

The primary objective of this systematic review was to evaluate the diagnostic accuracy of CRP, PCT, and WBC count in detecting early post-surgical infections across various surgical disciplines. The findings indicate that CRP remains a widely used and effective biomarker for early infection detection, with its diagnostic accuracy varying depending on the type of surgery and the POD on which it is measured. Adamina et al. [14] demonstrated that CRP levels on POD 4 had the highest accuracy (AUC = 0.76) in ruling out infectious complications after abdominal surgeries, with a negative predictive value (NPV) of 84.3%. Similarly, Plat et al. (2021) confirmed the utility of CRP monitoring on PODs 3, 4, and 5, identifying cut-off values (175 mg/L, 130 mg/L, and 144 mg/L, respectively) that correlated with major infectious complications, thereby supporting CRP-based discharge decisions. However, Singh et al. [17] challenged the assumption that investigating rising CRP levels leads to earlier infection detection, as their study found that elevated CRP on PODs 3-5 did not significantly improve clinical outcomes. These findings underscore the importance of CRP as a reliable screening tool but also highlight its limitations in guiding clinical decision-making when used in isolation.

Beyond CRP, alternative biomarkers such as PCT and NLR have emerged as potential adjuncts for infection monitoring. Cossé et al. [19] demonstrated that a PCT-based algorithm improved decision-making in adhesion-related small bowel obstruction, leading to shorter hospital stays and reduced time to surgery. Similarly, Peng et al. [16] found that NLR had moderate diagnostic accuracy (AUC = 0.80) in orthopedic surgeries, reinforcing the need for biomarker combinations rather than reliance on a single marker. Notably, in spinal surgery, Kang et al. [20] reported CRP as a highly sensitive predictor (100%) of surgical site infections, with a second rise in CRP levels on POD 5-7 indicating a high-risk infection scenario. In contrast, Warschkow et al. [21] observed moderate diagnostic accuracy of CRP in pancreatic surgeries, with higher sensitivity occurring later (POD 6-7) compared to abdominal procedures (POD 4-5). These findings emphasize that the diagnostic utility of CRP and other biomarkers is highly dependent on surgical type, postoperative timing, and patient-specific factors, reinforcing the need for individualized, multi-biomarker approaches to optimize early infection detection and intervention.

The findings of this systematic review have significant clinical implications for surgeons, infection control teams, and perioperative care providers in optimizing early detection and management of post-surgical infections. CRP remains a valuable biomarker, particularly in abdominal and colorectal surgeries, where studies have established cut-off values on PODs 3-5 to guide clinical decision-making [22]. Elevated CRP levels beyond 130-175 mg/L on these days have been associated with a higher risk of infectious complications, supporting its use as a discharge criterion or an indicator for further evaluation [23]. However, misinterpretation of CRP trends, such as assuming a single elevated reading equates to infection, can lead to unnecessary imaging, prolonged hospitalization, and antibiotic overuse, contributing to antimicrobial resistance. In orthopedic surgeries, the NLR has shown moderate accuracy (AUC = 0.80) in predicting infection, but its variability across different surgical settings suggests it should be used alongside other markers for better reliability. PCT, as seen in ASBO, has demonstrated utility in reducing hospital stays and unnecessary surgeries, reinforcing its role in triaging high-risk patients and guiding intervention timing. Despite these advantages, false negatives remain a clinical concern, particularly in patients with pre-existing inflammatory conditions or immunosuppression, where biomarker fluctuations may not reliably indicate infection status. To mitigate these risks, a multi-biomarker, algorithm-based approach, incorporating CRP, PCT, NLR, and clinical findings, is essential for precise infection detection, appropriate antibiotic stewardship, and improved patient outcomes across various surgical specialties.

The findings of this systematic review align with previous meta-analyses and clinical trials that have established CRP as a primary biomarker for detecting early post-surgical infections, particularly in abdominal and colorectal surgeries. Studies such as those by Warschkow et al. [21] and Adamina et al. [14] corroborate that CRP levels on POD 4-7 exhibit moderate to high diagnostic accuracy, with negative predictive values exceeding 80%, supporting its role in guiding clinical decision-making and discharge protocols. Similarly, PCT has been validated in previous literature as an effective early marker, particularly in critically ill patients, and its utility in ASBO management, as shown by Cossé et al. [19], further reinforces its role in reducing unnecessary surgical interventions and optimizing hospital stays. However, guidelines such as those from the WHO, CDC, and ERAS protocols currently do not endorse specific CRP or PCT cut-off values for universal use, instead emphasizing a comprehensive clinical evaluation alongside biomarker trends. By contrast, this review highlights the importance of standardized thresholds, particularly in high-risk procedures, to improve diagnostic accuracy and early intervention strategies. Furthermore, biomarker trends vary significantly across surgical disciplines, with NLR demonstrating moderate predictive value (AUC = 0.80) in orthopedic surgeries, whereas CRP shows superior accuracy in spinal and abdominal surgeries. These findings suggest that a surgical field-specific biomarker approach, rather than a one-size-fits-all model, may be more effective in post-surgical infection surveillance, potentially informing future updates to clinical guidelines.

This systematic review has several strengths, including a rigorous study selection process that adhered to standardized quality assessment tools such as AMSTAR 2, ROB 2, and QUADAS-2, ensuring a comprehensive and unbiased evaluation of the included studies. By incorporating a comparative analysis of multiple biomarkers (CRP, PCT, WBC, and NLR) across diverse surgical fields, this review provides a broader perspective on post-surgical infection detection than previous single-biomarker studies. Furthermore, the inclusion of recent clinical trials and meta-analyses enhances the relevance and applicability of findings to current surgical practices, making this review a valuable resource for evidence-based perioperative infection management. However, several methodological limitations must be acknowledged. The heterogeneity in study designs, with both retrospective and prospective studies included, introduces variability in outcomes, potentially affecting the consistency of findings. In addition, biomarker cut-off values and diagnostic criteria differed across studies, limiting direct comparability. Some included RCTs had small sample sizes, reducing statistical power, and differences in infection definitions (mild vs. severe) could have influenced sensitivity and specificity estimates. Publication bias is another concern, as studies with negative or inconclusive results may be underrepresented, and cohort studies may have selection bias due to non-randomized patient inclusion. To address these challenges, future research should prioritize large-scale, multi-center RCTs with standardized biomarker thresholds and uniform infection definitions, ensuring greater generalizability and clinical applicability of findings in post-surgical infection surveillance.

The findings of this systematic review support the development of a biomarker-driven infection monitoring algorithm tailored to different surgical fields [24]. For abdominal and colorectal surgeries, CRP levels on PODs 3-5 can be used as a discharge criterion, with thresholds below 130 mg/L supporting safe early discharge. In spinal and orthopedic surgeries, serial CRP and NLR measurements can help identify high-risk patients requiring closer monitoring. PCT-based protocols in cases of small bowel obstruction and sepsis-prone procedures can aid in antibiotic de-escalation, reducing unnecessary antibiotic exposure and antimicrobial resistance [25]. Unlike traditional infection detection methods that rely on clinical symptoms or imaging, serial biomarker monitoring offers a dynamic, cost-effective approach that can minimize the need for repeated imaging, invasive microbiological cultures, and prolonged hospital stays. By incorporating biomarker trends rather than isolated values, clinicians can achieve earlier intervention, reduced overtreatment, and optimized resource allocation in post-surgical care.

Despite the promising role of biomarkers in infection detection, several knowledge gaps remain. The optimal biomarker cut-offs for different surgical specialties require validation through large, multi-center RCTs, particularly in distinguishing mild inflammatory responses from true infections. Future research should explore multi-biomarker models, combining CRP, PCT, and NLR to enhance predictive accuracy across surgical disciplines. Additionally, artificial intelligence (AI) and machine learning algorithms could be integrated into clinical workflows to analyze biomarker trends in real time, improving early risk stratification and individualized treatment plans. Investigating genetic predispositions and patient-specific factors that influence postoperative inflammatory responses could further refine infection prediction models. Addressing these gaps will pave the way for personalized, biomarker-driven infection surveillance strategies, enhancing surgical outcomes and patient safety on a global scale.

## Conclusions

This systematic review highlights the diagnostic utility of CRP, PCT, and NLR in detecting early post-surgical infections, emphasizing their clinical relevance across various surgical specialties. By integrating biomarker-based monitoring into routine post-surgical care, clinicians can enhance infection detection, optimize patient management, and reduce unnecessary imaging and antibiotic overuse, ultimately improving patient safety and healthcare cost-effectiveness. The findings support a multi-modal approach, where biomarkers serve as an initial screening tool, complemented by clinical evaluation, imaging, and microbiological confirmation when necessary, ensuring a balanced and evidence-based strategy for infection surveillance. Moving forward, establishing standardized biomarker thresholds, conducting large-scale RCTs, and leveraging AI-driven predictive models will be key to refining postoperative infection management and risk stratification, paving the way for personalized, data-driven surgical care.
